# Supplementary material for: Structural shifts in China’s oil and gas CH4 emissions with implications for mitigation efforts
Source: Nat Commun. 2025 Mar 25;16:2926. doi: 10.1038/s41467-025-58237-z (PMC11937328; doi:10.1038/s41467-025-58237-z)
Supplement: Supplementary file 1 — Supplementary Information [file 41467_2025_58237_MOESM1_ESM.pdf]

Supplement Information for

Structural shifts in China's oil and gas CH_4 emissions and implications for mitigation efforts

Junjun Luo[1], Helan Wang[1], Hui Li[1], Bo Zheng[1,2]*

[1]Shenzhen Key Laboratory of Ecological Remediation and Carbon Sequestration, Institute of Environment and Ecology, Tsinghua Shenzhen International Graduate School, Tsinghua University, Shenzhen 518055, China.

[2]State Environmental Protection Key Laboratory of Sources and Control of Air Pollution Complex, Beijing 100084, China.

*Correspondence to: Bo Zheng (bozheng@sz.tsinghua.edu.cn)

Supplementary Discussion 1: The comparison of emission trends estimated using local emission factors with those using IPCC values and those in other bottom-up inventories

This study developed a set of emission factors specifically suited to the conditions of provinces or cities in China to estimate annual CH_4 emissions from the oil and gas industry. We employed the same calculation method and activity data but adopted the IPCC default emission factors (EFs) to assess the impact of the local EFs in this study on the emission estimates. The emission trends estimated using the IPCC default EFs compare well with the Emissions Database for Global Atmospheric Research (EDGARv8.0[1]), China's national inventory reported to the United Nations Framework Convention on Climate Change (UNFCCC)[2], and Liu et al[3] from 1990 to 2000. However, the emission trends with local EFs are higher than those derived from the IPCC EFs, as well as those reported by EDGARv8.0, UNFCCC, and Liu et al. This is mostly due to the substantial increase in emissions from the Shaanxi province.

Shaanxi's CH_4 emissions from the oil and gas systems surged more than 400-fold, rising from 2.9 $GgCH_4$ yr^{-1} in 1990 to 1207.2 $GgCH_4$ yr^{-1} in 2022 (Supplementary Figs. 1 and 2). The province's contribution to national total emissions escalated dramatically from 0.7% in 1990 to 30% in 2022. Most of the increase occurred after the 2000s with a total increase of 1154 $GgCH_4$ between 2000 and 2022, which accounted for 96% of the overall increase over the past three decades. This pronounced growth observed after 2000 coincided with the substantial increase in local gas production, mostly from unconventional gas-tight gas, driven by China's policy to enhance gas production capacity. Shaanxi encompasses China's major tight gas-producing fields, such as Sulige, Daniudi, and Jingbian, which together contribute to more than 90% of the province's total gas production. Compared with conventional gas, extracting tight gas from low-permeability formations requires energy-intensive processes, such as horizontal drilling and hydraulic fracturing, which lead to additional emissions. Moreover, during the extraction process, there is a longer flow-back period associated with well completion and workover, resulting in fugitive emissions. Consequently, these tight gas-producing fields have much higher CH_4 emission factors that can be even ten times higher than those of conventional gas fields[4]. The high emission factors of tight gas-producing fields have been considered in the estimation of the Shaanxi-specific emission factor in this study, which likely contributes to the discrepancies in emissions trends calculated using local emission factors compared to those derived from IPCC EFs and other studies.

Supplementary Discussion 2: The comparison of emission values with bottom-up inventories and top-down studies

CH_4 estimates in our study are lower than the estimates of Liu et al.[3] and EDGARv8.0[1], before 2010, but close to both inventories after 2010 (Supplementary Fig. 3). Our estimated figures are notably higher than the U.S. Environmental Protection Agency (EPA, https://cfpub.epa.gov/ghgdata/nonco2/), Global Fuel Exploitation Inventory (GFEI[5]), and UNFCCC[2]. For China, both EPA and GFEI[5] relied on data primarily sourced from National Communications and Biennial Update Reports reported to the UNFCCC[2], thus similar magnitudes and trends of emissions were estimated in these three datasets. The large discrepancy between our study and these inventories is possibly due to inconsistent accounting boundaries and the uncertainty of emission factors used[6-8]. For example, the major activity data applied in UNFCCC is the consumption of oil and natural gas[2] instead of the production and pipeline length applied in our study, resulting in the different calculation boundaries and the use of varying emission factors that can differ by more than an order of magnitude[6, 7].

The comparisons of our estimated figures with the top-down (TD) studies relying on atmospheric observations within an inverse-modeling framework show that our results fall within the range of the TD studies conducted between 2010 and 2019 (Supplementary Table 1). CH_4 emissions inferred from satellite remote sensing methods vary substantially, ranging from 0.7 to 5.9 $TgCH_4$ yr^{-1}[9-13]. This variability may be attributed to prior flux estimates, observation means, and sectoral partitioning[14-16]. Miller et al.[11] and Zhang et al.[13] applied EDGAR as a prior inventory, while other TD studies[9, 10, 12] above used GFEI or UNFCCC, potentially leading to lower emission estimates. In addition, satellite-based observation could contain a wide range of uncertainties and sectoral partitioning could be problematic in the TD method because of the uncertainty in the percentage of the oil and gas systems in the total CH_4 budget[14].

Supplementary Discussion 3: Stronger mitigation potential in upstream facilities

We assessed infrastructure-specific opportunities for mitigating CH_4 emissions based on our comprehensive emission dataset. Our findings indicate that targeting a small percentage of high-emitting facilities can disproportionately reduce CH_4 emissions of the oil and gas systems. Nationally, more than 60% of CH_4 emissions in 2022 from the oil and gas industry were generated by only 10% of the total oil and gas infrastructure. Of these high-emitting facilities, 76% were onshore fields, accounting for 58% of the national total CH_4 emissions from the oil and gas sectors in 2022. Therefore, it is essential to target emissions from these upstream facilities. The significance of high-emitting onshore fields is particularly striking in Shaanxi province (Supplementary Fig. 4), where 11 fields (8% of the top 10% of emitting facilities) produced 28% of the national total CH_4 emissions in 2022.

Supplementary Discussion 4: The transition of CH_4 emission from town gas to natural gas in the gas distribution segment

From 1990 to 2000, under policies such as the "Seventh Five-Year Plan for Town Gas Development" and the "Technical Policy for Town Gas Development", CH_4 emissions from gas distribution in Chinese cities were primarily driven by town gas. During this decade, CH_4 emissions from town gas accounted for 65% of total emissions from distribution pipelines on average (Supplementary Fig. 5). In contrast, CH_4 emissions from natural gas distribution pipelines represented only 35%, and the growth in these emissions was concentrated in a few specific cities, particularly the four municipalities directly under the central government. Specifically, in Beijing, the growth rate of CH_4 emissions from natural gas distribution pipelines during the 1990-2000 period was notable, with an annual increase of approximately 0.2 $GgCH_4\ yr^{-1}$. This rise is largely attributed to the commissioning of the Shaanxi-Beijing natural gas transmission pipeline in 1997, transporting natural gas resources from Shaanxi, Gansu, and Ningxia to Beijing.

The shift in CH_4 emissions in the gas distribution segment from town gas to natural gas has been widely observed in cities since 2000 (Supplementary Fig. 5). It is primarily driven by the high costs, poor quality, and environmental pollution issues associated with town gas, leading to its gradual replacement by cleaner and more affordable natural gas in urban gas supply systems. From 2001~2011, 289 cities nationwide showed positive trends in CH_4 emissions from natural gas distribution pipelines. In contrast, 81 cities demonstrated negative trends in CH_4 emissions from town gas, particularly in eastern coastal metropolitan areas such as Shanghai, Beijing, and Tianjin.

Benefiting from a series of natural gas development plans implemented after the Twelfth Five-Year Plan, this transition was even more pronounced from 2012 to 2022. During this period, the increase in CH_4 emissions from natural gas distribution pipelines in 185 cities was twice that observed from 2001 to 2011. Moreover, in 40 cities, CH_4 emissions from town gas distribution pipelines shifted from gains to losses compared to 2001-2011, mainly located in northern China (Supplementary Figs. 5e and 5f). Notably, Shanghai experienced robust emission reduction from town gas distribution during 2012-2022, decreasing emissions by 2 Gg over the ten years and successfully transitioning to the full utilization of natural gas.

Supplementary Discussion 5: Provincial emission transfer embodied in the production-consumption mismatch of oil and gas resources

The emission hotspots have been identified in major gas-producing provinces in the western regions of China, such as Shaanxi, Xinjiang, and Sichuan, rather than in the densely populated and economically developed eastern areas (Supplementary Fig. 7a). This is largely attributed to the remarkable emissions from production-field sources, which are predominantly located in western China. However, the primary consumption centers are primarily located in the eastern regions, indicating the spatial unevenness between production and consumption in China's oil and gas industry. This production-consumption imbalance leads to the transfer of upstream CH_4 emissions from eastern downstream provinces to western upstream provinces. Specifically, in resource-abundant but low-consumption provinces, like Shaanxi, Xinjiang, and Sichuan, upstream CH_4 emissions (i.e., those from field sources) accounted for more than 70% of the total upstream emissions in China, while these provinces contributed only about 10% to the nation's total oil and gas consumption (Supplementary Fig. 7b). In contrast, many coastal and central provinces with limited oil and gas resources show lower upstream emissions but have high consumption for these resources. For instance, in 2022, Shandong accounted for only approximately 2% of national upstream emissions, but its consumption of oil and gas, 164.4 Tg of standard oil (with natural gas converted to crude oil equivalent based on calorific value), surpasses 16% of the national consumption, making it the highest in the country.

Supplementary Table 1 Comparison of CH_4 emissions from the oil and gas systems in China with recent top-down studies.

Top-down studies	Observation	Prior inventory	Study year(s)	Emissions (Tg yr^{-1})	This work (Tg yr^{-1})
Miller et al.[11]	GOSAT	EDGAR v4.2	2010-2015	5.9	2.2
Zhang et al.[13]	GOSAT+Surface	EDGAR v4.3.2	2010-2017	2.4	2.4
Lu et al.[10]	GOSAT	GFEI v1	2010-2017	0.7	2.4
Worden et al.[12]	GOSAT	GFEI v1	2019	1.2	3.2
Chen et al.[9]	TROPOMI	UNFCCC	2019	2.6	3.2

Supplementary Table 2 The activity data and its multisource information in this study.

Emission segment	Activity data	Data source
Oil exploration & production	Onshore oil production volume	Province-level: China's National Bureau of Statistics (https://www.stats.gov.cn/)<br>City-level: Statistical Yearbook for 50 major oil-producing cities (e.g., Dongying Statistical Yearbook 2022[17]); Economic Census Yearbook for Guangdong, Henan, and Jiangsu provinces[18-20]; EPS data platform (https://www.epsnet.com.cn)
	Offshore oil production volume	China Land & Resources Almanac[21]
Oil transport	Oil transport volume	China's National Bureau of Statistics
Oil refining	Oil refining volume	China's National Bureau of Statistics
Gas exploration & production & processing	Onshore conventional and unconventional gas production volume	Province-level: China's National Bureau of Statistics<br>City-level: Statistical Yearbook for 16 major gas-producing cities (e.g., Yulin Statistical Yearbook 2021[22]); China Economic Yearbook[23]; Economic Census Yearbook for Jilin, Inner Mongolia, Shanxi, and Sichuan provinces[24-27]; EPS data platform (https://www.epsnet.com.cn)
	Offshore gas production volume	China Land & Resources Almanac[21]
Gas transmission	Length of transmission pipeline	Country-level: China Statistical Yearbook[28], Natural Gas Development Report[29], Medium - and Long-Term Oil and Gas Pipeline Network Planning[30]<br>Pipeline-level: Official, public, and corporate documents for 247 gas transmission pipelines (e.g., Natural gas pipeline transportation cost-related information table[31])
Gas storage	Gas consumption volume	China's National Bureau of Statistics
Gas import/export	Number of LNG station	Global Energy Monitor (https://globalenergymonitor.org/)
Gas distribution	Length of natural gas and town gas distribution pipeline	Province-level: China's National Bureau of Statistics<br>City-level: China Urban Construction Statistical Yearbook[32]

Supplementary Table 3 The spatial resolution of activity data for different bottom-up inventories.

Activity data	This work	Peng et al., 2016[33]	Liu et al., 2021[3]	Schwietzke et al., 2014[34]	Höglund-Isaksson et al., 2017[35]	Scarpelli et al., 2020 and 2022[5, 36]
Oil production volume	Province/city	Province	Province	Country	Country	Country
Oil transport volume	Country	Unclear	Unclear	Unclear	Not calculated*	Country
Oil refining volume	Province	Unclear	Unclear	Unclear	Not calculated	Country
Gas production volume	Province/city	Province	Province	Country	Country	Country
Length of transmission pipeline	Pipeline	Not used*	Not used	Not used	Not used	Not used
Gas consumption volume	Province	Not used	Not used	Not used	Unclear	Country
Number of LNG station	Point	Not calculated	Not calculated	Not calculated	Not calculated	Not calculated
Length of distribution pipeline	City	Not used	Not used	Not used	Not used	Not used

*Note: Not calculated refers to the exclusion of the corresponding segment from emissions estimation. Not used refers to including this segment for estimation with alternative activity data instead of the activity data presented in the table. For example, the previous studies referenced in this table did not use the length of the transmission pipeline as the activity data to estimate emissions from the gas transmission segment but rather relied on other data, such as the consumption volume. However, according to IPCC guidelines, the length of the transmission pipeline is considered the best indicator of CH_4 emissions from this segment.

Supplementary Table 4 Emission factors for different emission segments in this study.

Emission segment	Activity data	Emission factor	Data source
Oil exploration & production	Onshore oil production	Field-specific	Masnadi et al[37].
	Offshore oil production	Field-specific	
Oil transport	Oil transport volume	Country-specific: 0.0061 kg m^{-3}	Official inventory[38]
Oil refining	Oil refining volume	Country-specific: 0.07 kg m^{-3}	
Gas exploration & production & processing	Onshore conventional production	Field-specific	Gan et al[4].
	Coalbed gas production	Field-specific	
	Offshore gas production	Field-specific	
Gas transmission	Length of transmission pipeline	3.09 kg m^{-1}	IPCC, 2019[39]
Gas storage	Gas consumption volume	0.48 g m^{-3}	
Gas import/export	Number of LNG station	1660 t $station^{-1}$	
Gas distribution	Length of natural gas pipeline	0.7 kg m^{-1}	
	Length of town gas pipeline	0.58 kg m^{-1}	

Supplementary Table 5 CH_4 emission factors for different oil production fields.

Field name	Field type	Exploration (kg m^{-3})	Production (kg m^{-3})
Huizhou 21-1	Offshore	0.005	24.18
Qinhuangdao 32-6	Offshore	0.002	1.40
Bozhong	Offshore	0.01	5.67
Ansai	Onshore	0.55	1.39
Jingan	Onshore	0.55	1.29
Karamay	Onshore	0.55	1.71
Lamadian	Onshore	0.55	1.49
Saertu	Onshore	0.55	1.59
Tahe	Onshore	0.56	1.58
Xingshugang	Onshore	0.55	1.65
Jiyuan	Onshore	0.55	1.40
Penglai 19-3	Offshore	0.002	0.87
Suizhong 36-1	Offshore	0.003	0.98
Fengcheng	Onshore	0.65	8.15

Supplementary Table 6 CH_4 emission factors for different gas production fields.

Field name	Field type	Exploration & Production (g m^{-3})	Processing (g m^{-3})
Shuangyushi&Jiulongshang	Onshore conventional	0.20	0.97
Anyue	Onshore conventional	0.03	1.05
Datianchi	Onshore conventional	1.06	0.80
Wolonghe	Onshore conventional	0.78	1.19
Mahe	Onshore conventional	1.28	0.89
Kelameili	Onshore conventional	1.65	0.43
Qingshen	Onshore conventional	1.22	0.92
Zhongba	Onshore conventional	1.33	0.86
Sebei	Onshore conventional	1.31	0.82
Tainan	Onshore conventional	1.33	0.82
Kekeya	Onshore conventional	1.33	0.92
Dongping	Onshore conventional	1.23	0.83
Luojiazhai	Onshore conventional	0.00	0.23
Dukouhe	Onshore conventional	1.54	0.87
Longgang	Onshore conventional	0.00	1.51
Tieshanpo	Onshore conventional	0.00	0.55
Hetianhe	Onshore conventional	1.57	1.13
Yuanba	Onshore conventional	4.07	18.19
Puguang	Onshore conventional	1.22	0.00
Dina2	Onshore conventional	2.58	3.97
Kela	Onshore conventional	0.97	0.86
Yingmai7	Onshore conventional	1.29	0.93
Tahe	Onshore conventional	1.02	0.09
Tazhong	Onshore conventional	1.09	0.01
Changling&Songnan	Onshore conventional	0.47	0.00
Sulige	Onshore tight gas	12.06	0.80
Guangan	Onshore tight gas	15.66	0.76
Yingtai	Onshore tight gas	14.75	0.09
Hechuan	Onshore tight gas	13.31	1.44
Yulin	Onshore tight gas	16.56	0.96
Zhaotong	Onshore tight gas	17.25	0.76
Daniudi	Onshore tight gas	16.72	0.79
Bajiaochang	Onshore tight gas	18.64	0.78
Changning&Weiyuan	Onshore shale gas	16.23	0.83
Wushenqi	Onshore tight gas	16.27	1.66
Jingbian	Onshore tight gas	20.06	0.00
Yanchang	Onshore shale gas	19.95	0.30
Mizhi	Onshore tight gas	19.01	0.78
Zizhou	Onshore tight gas	15.95	1.31
Shenmu	Onshore tight gas	20.89	0.75
Xinchang	Onshore tight gas	14.24	0.80

Luodai	Onshore tight gas	17.62	0.81
Fuling	Onshore shale gas	21.39	0.79
Dabei	Onshore tight gas	16.74	0.88
Keshen	Onshore tight gas	17.01	0.89
Juggar CBM	Coalbed methane	4.05	0.83
Qinshui CBM	Coalbed methane	6.71	0.79
Bishuixing CBM	Coalbed methane	7.08	0.83
Ordos CBM	Coalbed methane	10.48	0.91
Chunxiao	Offshore	0.25	0.74
Liwan	Offshore	1.31	0.71
Panyu	Offshore	1.59	0.63
Lingshui	Offshore	1.92	1.46
Bozhong	Offshore	1.80	0.21
Ya	Offshore	2.16	1.17
Wenchang	Offshore	0.45	0.86
Ledong	Offshore	0.00	0.00
Dongfang	Offshore	0.73	1.18
Qiongxi	Offshore	14.76	0.76

Supplementary Table 7 Years of population and land use map for urban population grid data.

Population map (year)	Land use cover map (year)	Urban population map (version)	Emission allocation years
2000	1990	v1	1990~1994
2000	1995	v2	1995~1999
2000~2004	2000	v3~v7	2000~2004
2005~2009	2005	v8~v12	2005~2009
2010~2014	2010	v13~v17	2010~2014
2015~2020	2015	v18~v23	2015~2022

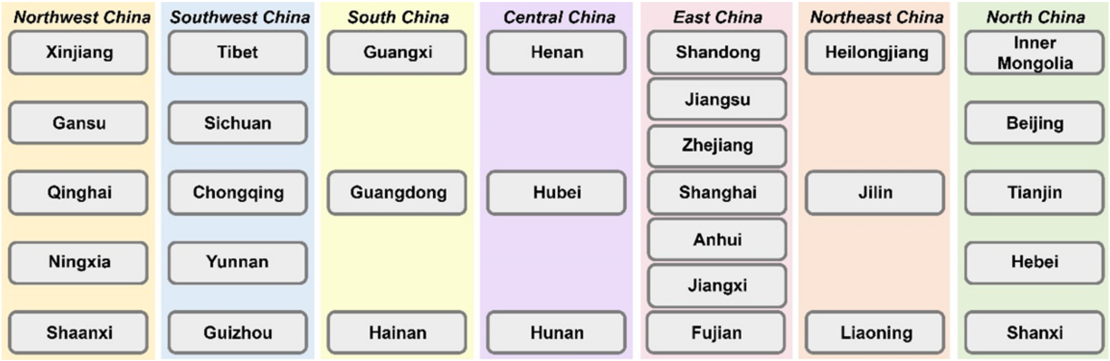

Supplementary Fig. 1 Region split in China. The gray boxes represent the 31 provinces of China.

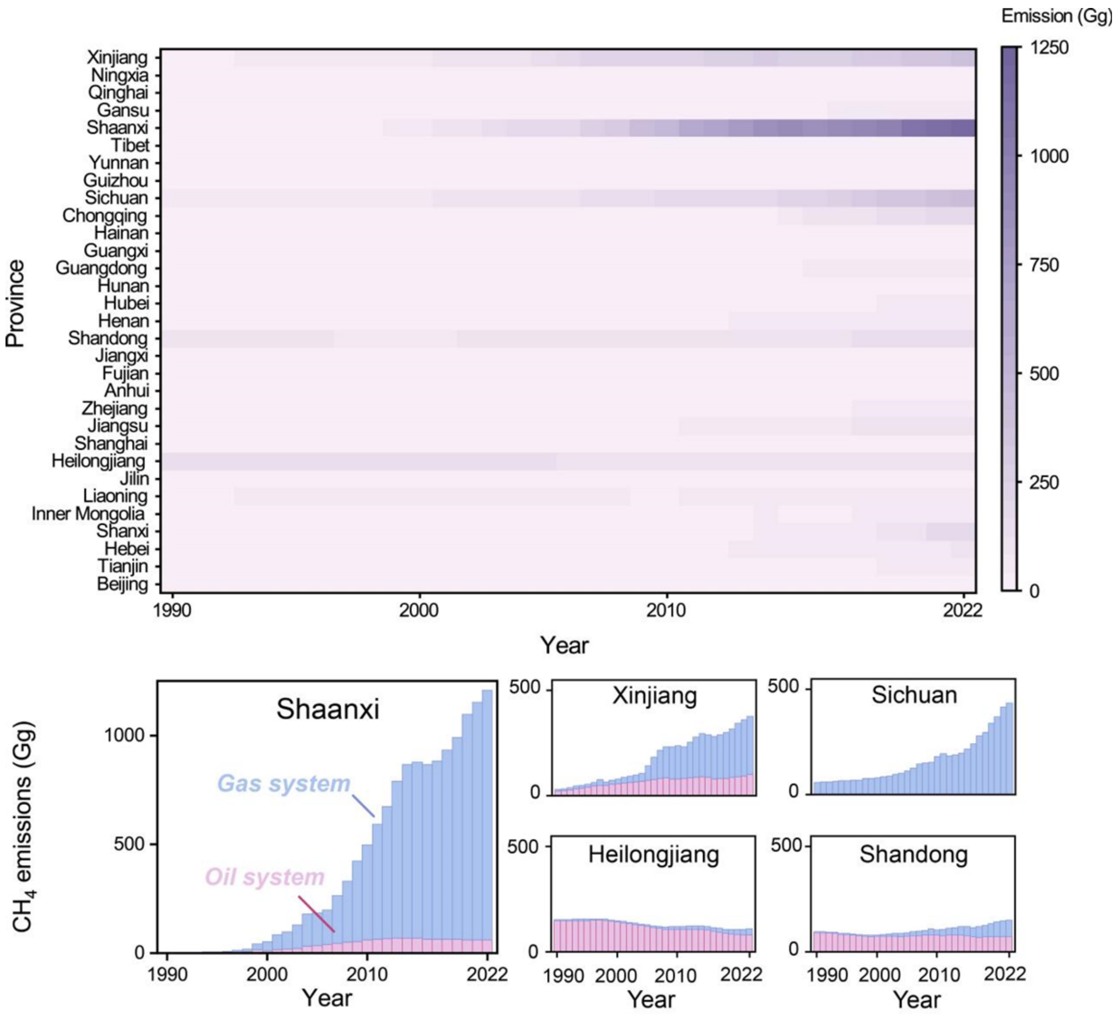

Supplementary Fig. 2 Variations in CH_4 emissions from the oil and gas sectors for 31 provinces from 1990 to 2022 and the emissions changes in typical provinces.

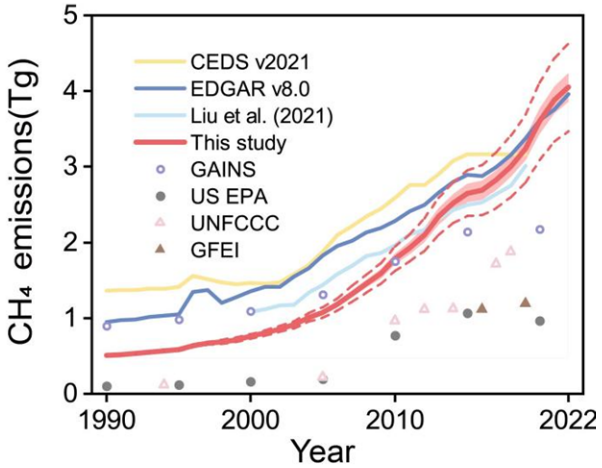

Supplementary Fig. 3 China's CH_4 emissions of the oil and gas systems from different bottom-up datasets. The emission datasets include CEDS v2021[40], EDGAR v8.0 (2023)[1], Liu et al[3], GAINS (http://gains.iiasa.ac.at/models), EPA (https://cfpub.epa.gov/ghgdata/nonco2/), UNFCCC[2], and GFEI[5]. The red-shaded area presents the 95 % confidence interval (CI) of our emission estimates. The two dashed curves present the sensitivity estimates of CH_4 emissions based on the limited and extensive implementation of lower-emitting technologies and practices respectively.

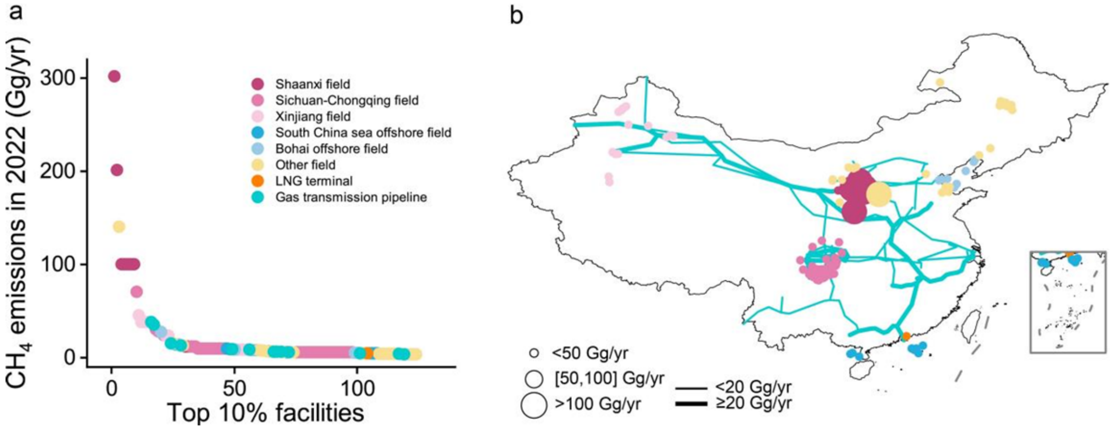

Supplementary Fig. 4 Top 10% of high-emission oil and gas facilities in 2022. (a) shows their CH_4 emissions, and (b) shows the spatial distribution of emissions from these facilities. Panel (b) was created using Python 3.9, utilizing the mpl_toolkits.basemap package to import Basemap 1.3.7[41].

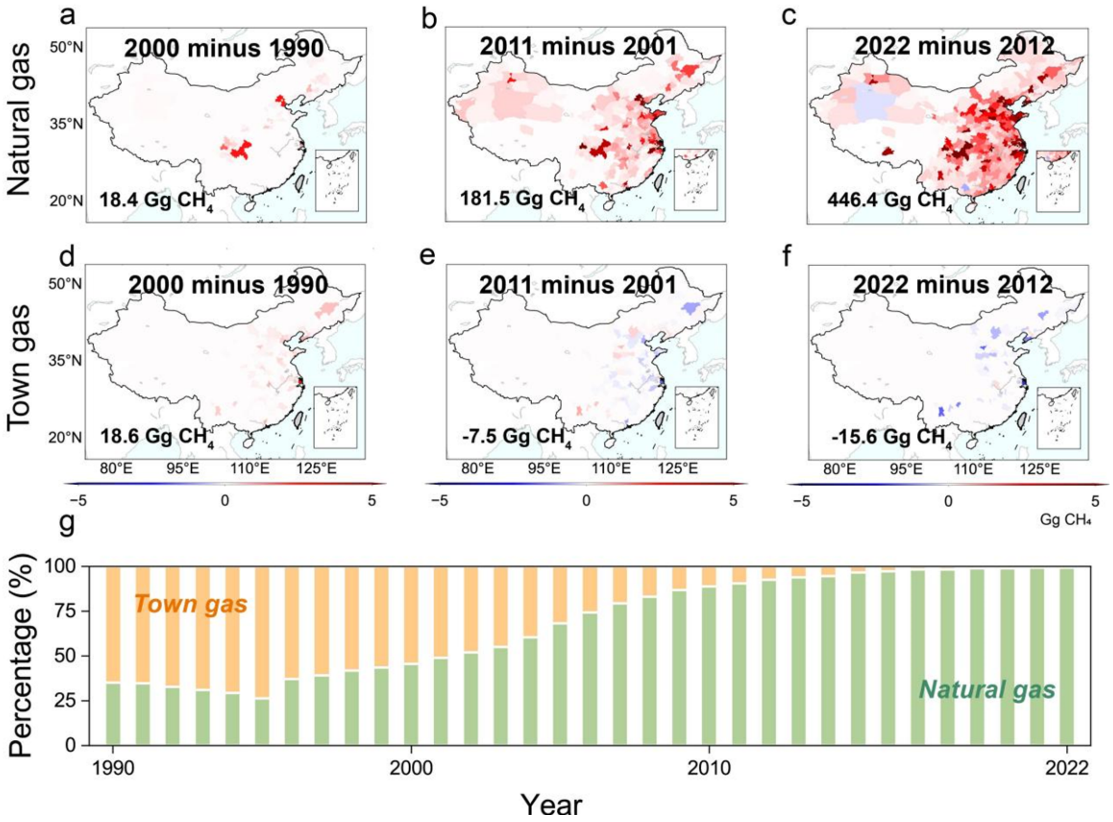

Supplementary Fig. 5 The changes in CH_4 emissions from the gas distribution segment in China during 1990–2022. The spatial distribution of emission differences from natural gas distribution (a) between 1990 and 2000, (b) 2001 and 2011, (c) 2012 and 2022, and the spatial distribution of emission differences from town gas distribution (d) between 1990 and 2000, (e) 2001 and 2011, (f) 2012 and 2022. (g) shows the changes in the proportion of CH_4 emissions from natural gas distribution pipelines versus town gas distribution pipelines from 1990 to 2022. Panels (a)-(f) were created using Python 3.9, utilizing the mpl_toolkits.basemap package to import Basemap 1.3.7[41].

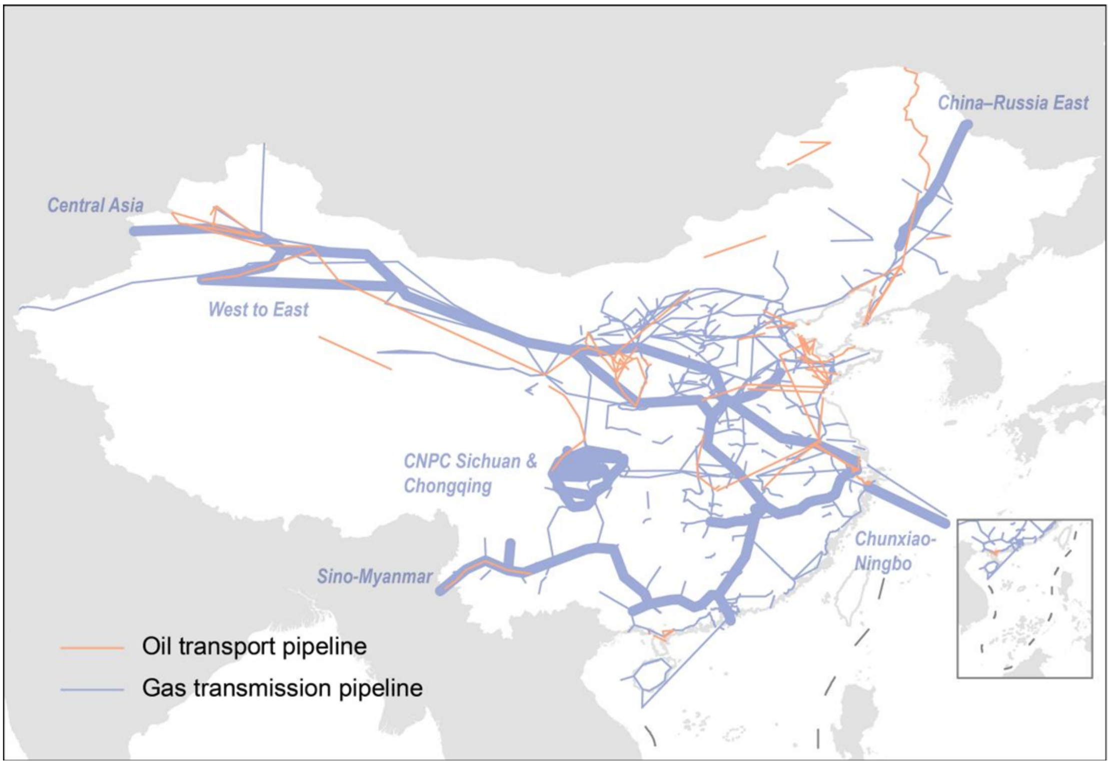

Supplementary Fig. 6 China's pipeline distribution map in operation in 2022. The bold lines indicate the main pipelines. This figure was created using Python 3.9, utilizing the mpl_toolkits.basemap package to import Basemap 1.3.7[41].

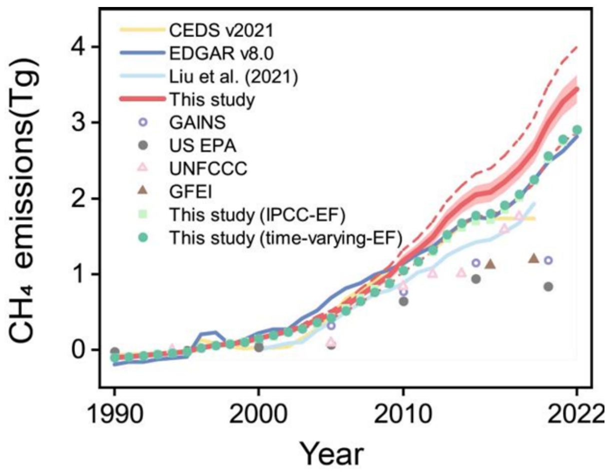

Supplementary Fig. 7 Comparison of annual CH_4 emissions relative to the average in China under different experiments and studies. This study indicates the estimates applying the local emission factors. This study (IPCC-EF) indicates the estimates using the same method and activity data but applying the IPCC emission factors. This study (time-varying-EF) indicates the estimates using the local upstream emission factors with a 1% annual reduction.

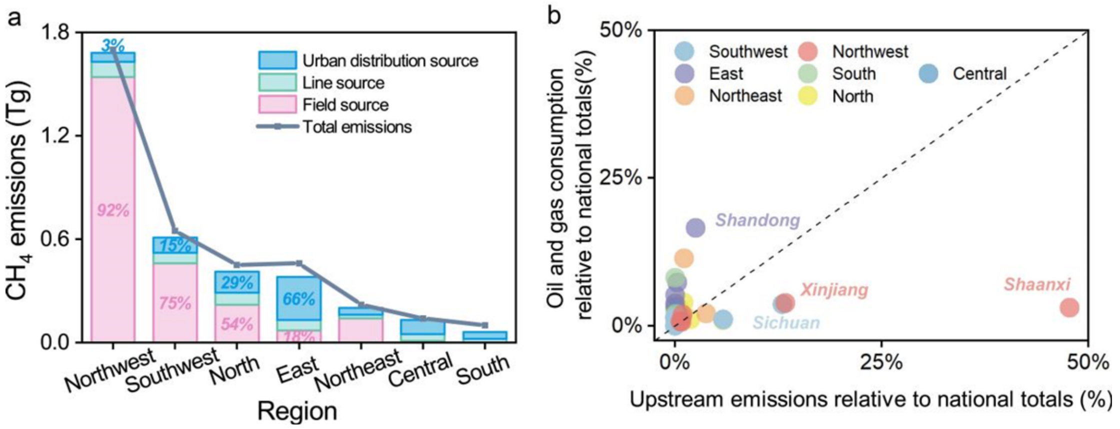

Supplementary Fig. 8 The transfer of upstream CH_4 emissions from eastern downstream provinces to western upstream provinces. (a) CH_4 emissions of the oil and gas systems by regions and sources and (b) the comparison of 31 provinces' respective upstream CH_4 emissions relative to national total emissions with their respective oil and gas consumption relative to national total consumption in 2022.

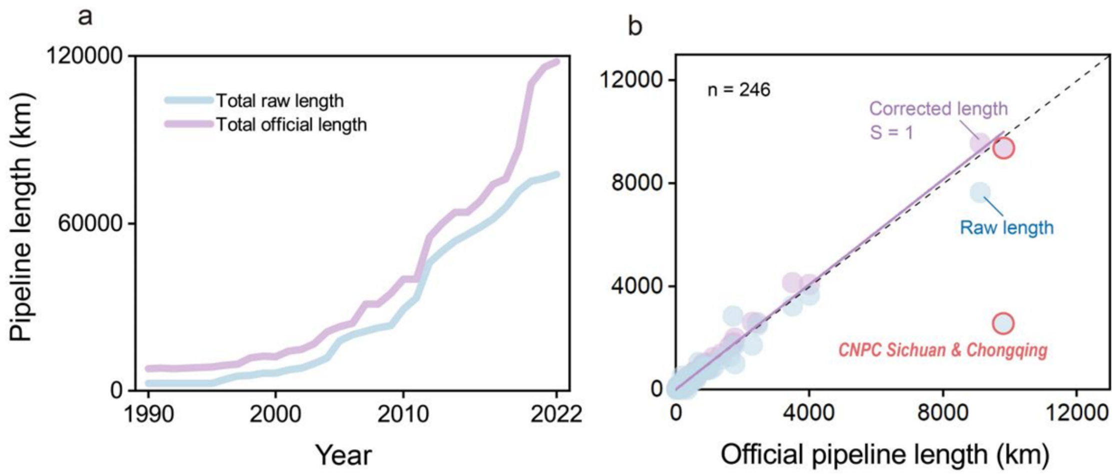

Supplementary Fig. 9 Comparison of the raw pipeline length, the official pipeline length, and the corrected pipeline length. (a) compares the total raw length and the officially reported total length from 1990 to 2022. (b) compares the official reported lengths (x-axis) of 246 pipelines with their respective lengths before and after reconstruction (y-axis). The blue circles refer to the comparison between the official lengths and the raw lengths. The purple circles indicate the comparison between the 1990 – 2022 average adjusted lengths with the official lengths. The red circles highlight the CNPC Sichuan & Chongqing Network, showing a significant deviation between its raw length and the recorded data. Its corrected length aligns closely with the official length with an error margin of only 4%.

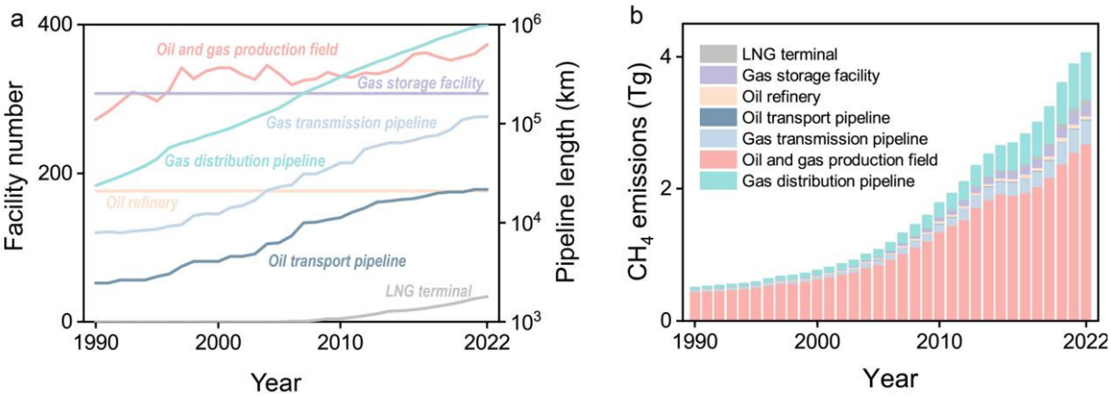

Supplementary Fig. 10 The number (or pipeline length) of the oil and gas infrastructure in this study and their CH_4 emissions from 1990 to 2022. Note that the oil transport pipelines, gas transmission pipelines, and gas distribution pipelines are presented by their annual total lengths, while other facilities are shown as their annual total numbers.

Supplementary Reference

1. Crippa M, *et al.* Insights into the spatial distribution of global, national, and subnational greenhouse gas emissions in the Emissions Database for Global Atmospheric Research (EDGAR v8.0). *Earth Syst Sci Data* **16**, 2811-2830 (2024).
2. Ministry of Ecology and Environment of China. Third Biennial Update Report on Climate Change of the People's Republic of China. (2023).
3. Liu G, *et al.* Recent Slowdown of Anthropogenic Methane Emissions in China Driven by Stabilized Coal Production. *Environmental Science & Technology Letters* **8**, 739-746 (2021).
4. Gan Y, *et al.* Carbon footprint of global natural gas supplies to China. *Nature Communications* **11**, 9 (2020).
5. Scarpelli TR, *et al.* Updated Global Fuel Exploitation Inventory (GFEI) for methane emissions from the oil, gas, and coal sectors: evaluation with inversions of atmospheric methane observations. *Atmospheric Chemistry and Physics* **22**, 3235-3249 (2022).
6. Saunois M, *et al.* The Global Methane Budget 2000–2017. *Earth System Science Data* **12**, 1561-1623 (2020).
7. Gao JL, Guan CH, Zhang B. Why are methane emissions from China's oil & natural gas systems still unclear? A review of current bottom-up inventories. *Sci Total Environ* **807**, 12 (2022).
8. Tibrewal K, *et al.* Assessment of methane emissions from oil, gas and coal sectors across inventories and atmospheric inversions. *Communications Earth & Environment* **5**, (2024).
9. Chen Z, *et al.* Methane emissions from China: a high-resolution inversion of TROPOMI satellite observations. *Atmospheric Chemistry and Physics* **22**, 10809-10826 (2022).
10. Lu X, *et al.* Global methane budget and trend, 2010–2017: complementarity of inverse analyses using in situ (GLOBALVIEWplus CH_4 ObsPack) and satellite (GOSAT) observations. *Atmospheric Chemistry and Physics* **21**, 4637-4657 (2021).
11. Miller SM, Michalak AM, Detmers RG, Hasekamp OP, Bruhwiler LMP, Schwietzke S. China's coal mine methane regulations have not curbed growing emissions. *Nature Communications* **10**, (2019).
12. Worden JR, *et al.* The 2019 methane budget and uncertainties at 1° resolution and each country through Bayesian integration Of GOSAT total column methane data and a priori inventory estimates. *Atmospheric Chemistry and Physics* **22**, 6811-6841 (2022).
13. Zhang YZ, *et al.* Observed changes in China's methane emissions linked to policy drivers. *Proc Natl Acad Sci U S A* **119**, 7 (2022).
14. Lu X, *et al.* Observation-derived 2010-2019 trends in methane emissions and intensities from US oil and gas fields tied to activity metrics. *Proceedings of the National Academy of Sciences* **120**, (2023).
15. Shen L, *et al.* National quantifications of methane emissions from fuel exploitation using high resolution inversions of satellite observations. *Nature Communications* **14**, (2023).
16. Varon DJ, *et al.* Continuous weekly monitoring of methane emissions from the Permian Basin by inversion of TROPOMI satellite observations. *Atmospheric Chemistry and Physics* **23**, 7503-7520 (2023).
17. Dongying City Statistics Bureau. *Dongying Statistical Yearbook*. China Statistics Press (2022).

18. Jiangsu provincial Bureau of Statistics. *Jiangsu Economic Census Yearbook*. China Statistics Press (2004).
19. Guangdong provincial Bureau of Statistics. *Guangdong Economic Census Yearbook*. China Statistics Press (2018).
20. Henan provincial Bureau of Statistics. *Henan Economic Census Yearbook*. China Statistics Press (2018).
21. Ministry of Land and Resources. *China Land & Resources Almanac* (2005-2018).
22. Yulin City Statistics Bureau. *Yulin Statistical Yearbook*. China Statistics Press (2021).
23. Development Research Center of the State Council. *China Economic Yearbook* (2010).
24. Jilin provincial Bureau of Statistics. *Jilin Economic Census Yearbook*. China Statistics Press (2008).
25. Inner Mongolia provincial Bureau of Statistics. *Inner Mongolia Economic Census Yearbook*. China Statistics Press (2018).
26. Shanxi provincial Bureau of Statistics. *Shanxi Economic Census Yearbook*. China Statistics Press (2018).
27. Sichuan provincial Bureau of Statistics. *Sichuan Economic Census Yearbook*. China Statistics Press (2018).
28. National Bureau of Statistics of China. *China Statistical Yearbook*. China Statistics Press (1990-2022).
29. National Energy Administration. *China Natural Gas Development Report*. Petrochina National high-end think tank research center (2016-2022).
30. National Development and Reform Commission of China. Medium - and Long-Term Oil and Gas Pipeline Network Planning. 2017, https://www.ndrc.gov.cn/xxgk/zcfb/ghwb/201707/t20170712_962238.html.
31. China National Petroleum Corporation. Natural gas pipeline transportation cost related information table. 2019, https://view.officeapps.live.com/op/view.aspx?src=https%3A%2F%2Fwww.cnpc.com.cn%2Fcnpc%2Ftrq2019%2F202005%2F91ae8e21f2b54b78a0bdd642e1bb9ca1%2Ffiles%2Ff58b5314e524486bb8ac2cb02178c03b.xlsx&wdOrigin=BROWSELINK.
32. National Bureau of Statistics of China. *China Urban Construction Statistical Yearbook*. Ministry of Housing and Urban-Rural Development of the People's Republic of China (MOHURD) (2000-2022).
33. Peng S, *et al.* Inventory of anthropogenic methane emissions in mainland China from 1980 to 2010. *Atmospheric Chemistry and Physics* **16**, 14545-14562 (2016).
34. Schwietzke S, Griffin WM, Matthews HS, Bruhwiler LMP. Global Bottom-Up Fossil Fuel Fugitive Methane and Ethane Emissions Inventory for Atmospheric Modeling. *ACS Sustainable Chemistry & Engineering* **2**, 1992-2001 (2014).
35. Höglund-Isaksson L. Bottom-up simulations of methane and ethane emissions from global oil and gas systems 1980 to 2012. *Environ Res Lett* **12**, (2017).
36. Scarpelli TR, *et al.* A global gridded (0.1° × 0.1°) inventory of methane emissions from oil, gas, and coal exploitation based on national reports to the United Nations Framework Convention on Climate Change. *Earth System Science Data* **12**, 563-575 (2020).
37. Masnadi MS, *et al.* Well-to-refinery emissions and net-energy analysis of China's crude-oil supply. *Nat Energy* **3**, 220-226 (2018).

38. National Development and Reform Commission of China. *National greenhouse gas inventory*. China Environmental Press (2014).
39. Buendia EC, Guendehou S, Limmeechokchai B, Pipatti R. 2019 Refinement to the 2006 IPCC Guidelines for National Greenhouse Gas Inventories.). Intergovernmental Panel on Climate Change (2019).
40. O'Rourke PR, Smith, S. J., Mott, A., Ahsan, H., McDuffie, E. E., Crippa, M., Klimont, S., McDonald, B., Z., Wang, Nicholson, M. B, Feng, L., and Hoesly, R. M. Community Emissions Data System (Version Feb-05-2021). 2021.
41. J. D. Hunter. Matplotlib: A 2D graphics environment. *Computing in Science & Engineering* **9**, 90-95 (2007).
